# 异长春花碱逆转肺癌顺铂耐药A549/DDP细胞耐药性的作用和机制

**DOI:** 10.3779/j.issn.1009-3419.2014.02.14

**Published:** 2014-02-20

**Authors:** 春胜 齐, 森 高, 会强 李, 卫真 高

**Affiliations:** 1 300070 天津，天津医科大学药理学教研室 Department of Pharmacology, Tianjin Medical University, Tianjin 300070, China; 2 300020 天津，中国医学科学院血液病医院，血液学研究所药剂科 Department of Pharmacy, Institute of Hematology, Blood Diseases Hospital Chinese Academy of Medical Sciences, Peking Union Medical College, Tianjin 300020, China; 3 300052 天津，天津医科大学总医院药剂科 Department of Pharmacy, Tianjin Medical University General Hospital, Tianjin 300052, China; 4 300070 天津，天津医科大学免疫学教研室 Department of Immunology, Tianjin Medical University, Tianjin 300070, China

**Keywords:** 异长春花碱, 肺肿瘤, 顺铂耐药, Vinorelbine, Lung neoplasms, Cisplatin resistance

## Abstract

**背景与目的:**

肺癌细胞耐药已经成为肺癌化疗的主要困难之一，异长春花碱被认为可有效抑制肺癌细胞的增殖和转移。本研究旨在探讨异长春花碱对人肺癌A549/DDP细胞顺铂耐受性的逆转作用及机制。

**方法:**

1 μmol/L和5 μmol/L异长春花碱作用A549/DDP细胞后，应用MTS法检测肿瘤细胞顺铂敏感性的变化，应用流式细胞术检测肿瘤细胞凋亡率变化，肿瘤细胞对Rh-123摄入量的变化，Western blot法检测MDR1、Bcl-2、survivin、caspase-3/8和PTEN蛋白表达以及Akt的磷酸化水平的变化，real-time PCR检测MDR1、Bcl-2、survivin和PTEN的mRNA表达，用报告基因系统检测NF-κB、Twist和Snail的转录活性。

**结果:**

1 μmol/L和5 μmol/L异长春花碱作用A549/DDP细胞后，肿瘤细胞对顺铂的敏感性分别提高了1.91倍和2.54倍，肿瘤细胞对Rh-123的摄入量提高了1.93倍和2.95倍，细胞凋亡增加了2.25倍和3.82倍，MDR1、Bcl-2、survivin蛋白表达和Akt磷酸化水平下调，caspase-3/8和PTEN蛋白表达上调，MDR1的mRNA表达下调43.5%和25.8%，Bcl-2的mRNA表达下调57.3%和34.1%，survivin的mRNA表达下调37.6%和12.4%，PTEN表达上调183.4%和154.2%，NF-κB转录活性下降53.2%和34.5%，Twist转录活性下降61.4%和33.5%，Snail转录活性下降57.8%和18.7%。

**结论:**

异长春花碱可提高肿瘤细胞A549/DDP对顺铂的敏感性，其机制可能与调节PTEN/AKT/NF-κB信号路径活性，进而下调耐药基因表达，上调促凋亡基因表达有关。

## Abstract (en)

**Background and objective:**

Drug resistance is a major obstacle on lung cancer treatment and Vinorelbine is an effective drug to inhibition of tumor proliferation and metastasis. In this study, we investigated the effect and mechanism of Vinorelbine on reversing the cisplatin resistance of human lung cancer A549/DDP cell line.

**Methods:**

With 1 μmol/L and 5 μmol/L Vinorelbine treatment, MTS assay was employed to determine the effect of the cisplatin sensitivity of tumor cells, flow cytometry to determine the apoptosis rate and change of Rh-123 content; Western blot to determine the expression of MDR1, Bcl-2, surviving, PTEN, caspase-3/8 and phosphorylation level of Akt (p-Akt); Real-time PCR was to determine the mRNA expression of MDR1, Bcl-2, survivin and PTEN. Finally the transcriptional activities of NF-κB, Twist and Snail were determined by reporter gene system.

**Results:**

With 1 μmol/L and 5 μmol/L Vinorelbine treatment, the sensitivity of cancer cells to cisplatin was increased by 1.91- and 2.54- folds respectively, flow cytometry showed that the content of Rh-123 was elevated 1.93- and 2.95- folds and apoptosis rate was increased 2.25- and 3.82- folds, Western blot showed that the expression of multidrug resistance related proteins MDR, Bcl-2 and survivin were downregulated, caspase-3/8 and PTEN was upregulated, phosphorylation of Akt was downregulated as well, real-time assay showed that the mRNA expression of MDR1 was downregulated 43.5% and 25.8%, Bcl-2 was downregulated 57.3% and 34.1%, survivin was downregulated 37.6% and 12.4%, PTEN was upregulated 183.4% and 154.2%, the transcriptional activities of NF-κB was downregulated 53.2% and 34.5%, Twist was downregulated 61.4% and 33.5%, and Snail was downregulated 57.8% and 18.7%.

**Conclusion:**

Vinorelbine treatment led to increase of cisplatin sensitivity of A549/DDP cells and the mechanisms included the regulation of PTEN/AKT/NF-κB signal pathway to decreased drug resistance gene expression and increased pro-apoptosis gene expression.

肺癌的治疗是以手术、放疗和化疗为主的综合治疗，化疗通过给予药物以杀死肿瘤细胞，一般与手术和放疗联合使用。顺铂是一种常见的化疗药物，可通过使DNA发生交联，抑制癌细胞的DNA复制和转录，使肿瘤细胞停止生长发生凋亡^[1]^。但临床实际中，肿瘤细胞往往对化疗药物产生耐受，使治疗失效。因此，查找肿瘤的耐药机制，开发新的逆转耐药性的方法，对提高临床患者的受益有十分重要的意义。目前的研究^[2, 3]^显示，肿瘤细胞产生耐药的机制包括减少药物的吸收，通过转运蛋白增加药物的外排，通过谷胱甘肽系统对抗肿瘤药物进行解毒，凋亡途径异常以减少肿瘤细胞的凋亡等。

异长春花碱（Vinorelbine）是一种新型抗肿瘤药，在一系列临床前研究中发现其可抑制肿瘤细胞的生长，促进细胞凋亡，抑制肿瘤转移^[4]^。而异长春花碱逆转顺铂耐受肺癌细胞的作用尚未得到充分研究，本研究拟对其作用和分子机制进行初步的探讨。

## 1 材料与方法

### 1.1 主要试剂与仪器

人肺癌细胞系A549和A549/DDP购自中国科学院上海生命科学研究院生物化学与细胞生物学研究所；细胞培养基购自Gibco公司；异长春花碱购自Sigma公司；顺铂购自Sigma公司；Rhodamine-123（Rh-123）购自Sigma公司；非放射性细胞增殖检测法（MTS）试剂、real-time PCR试剂盒购自Promega公司；细胞凋亡检测试剂盒购自BD Biosciences；抗MDR1（multidrug resistance protein 1）、Bcl-2、caspase-3/8、survivin、PTEN（Phosphatase and tensin homolog）和p-AKT单抗均购自SantaCruz公司；NF-κB、Twist和Snail的转录活性报告基因检测试剂盒购自Promega；ECL免疫印迹底物试剂盒购自Millipore公司；流式细胞仪CaliburTM：BD公司；酶标仪和PCR仪：Thermo公司。

### 1.2 细胞培养

A549和A549/DDP培养于10 cm培养皿，37 ℃、5%CO_2_和饱和湿度的培养箱中，培养基为90% EMEM，10%胎牛血清（FBS）。0.25%胰酶-EDTA消化传代，所有试验均采用对数生长期细胞。

### 1.3 MTS法检测异长春花碱对A549细胞的抑制作用

取对数生长期的转染克隆细胞，以（2-3）×10^4^个/mL接种到96孔微孔板中，100 μL/孔，培养过夜使细胞贴壁。向对应试验孔加入0.1 μmol/L、1 μmol/L、5 μmol/L、10 μmol/L、50 μmol/L、100 μmol/L异长春花碱，继续培养24 h，吸去培养基，按照试剂盒说明书加入MTS试剂，最后用酶标仪测定490 nm波长下的OD值，并计算药物对细胞的抑制率。抑制率＝（1-实验组OD值/对照组OD值）×100%。以异长春花碱浓度的对数为横坐标，抑制率为纵坐标作图并拟合抑制曲线，50%抑制率所对应的化合物浓度即为IC_50_值。

### 1.4 MTS法检测异长春花碱对A549/DDP细胞耐药性的逆转作用

取对数生长期的转染克隆细胞，以（2-3）×10^4^个/mL接种到96孔微孔板中，100 μL/孔，培养过夜使细胞贴壁。向对应试验孔加入0.1 μmol/L、1 μmol/L、5 μmol/L、10 μmol/L、50 μmol/L、100 μmol/L顺铂和1 μmol/L和5 μmol/L异长春花碱，继续培养72 h，吸去培养基，按照试剂盒说明书加入MTS试剂，最后用酶标仪测定490 nm波长下的OD值，并计算药物对细胞的抑制率。抑制率＝（1-实验组OD值/对照组OD值）×100%。以异长春花碱浓度的对数为横坐标，抑制率为纵坐标作图并拟合抑制曲线，50%抑制率所对应的化合物浓度即为IC_50_值。逆转倍数=异长春花碱处理组A549/DDP细胞IC_50_值/异长春花碱未处理组A549/DDP细胞IC_50_值。

### 1.5 流式细胞术检测A549/DDP细胞内Rh-123含量和细胞凋亡

取对数生长期的肿瘤细胞，1 μmol/L和5 μmol/L异长春花碱作用24 h后，加入10 μmol/L Rh-123染液，培养1 h后收集细胞，调整细胞浓度至10^6^/mL，以流式细胞仪检测细胞中Rh-123的荧光强度（488 nm激发光），考察异长春花碱对A549/DDP细胞中Rh-123含量的影响。

取对数生长期的肿瘤细胞，经10 μmol/L顺铂及1 μmol/L和5 μmol/L异长春花碱作用24 h后，加入PI和Annexin V-FITC各20 μL，避光孵育15 min后收集细胞，调整细胞浓度至10^6^/mL，以流式细胞仪检测细胞中PI和FITC的荧光强度（488 nm激发光），考察异长春花碱对A549/DDP细胞凋亡的影响。

### 1.6 Western blot法检测A549/DDP细胞MDR1、Bcl-2、caspase-3/8、survivin、PTEN和p-AKT蛋白的表达

取对数生长期的肿瘤细胞，1 μmol/L和5 μmol/L异长春花碱作用24 h后，收集细胞并裂解提取蛋白。BCA（bicinchoninic acid）法测定细胞裂解物的蛋白含量，取等量蛋白质以12% SDS-PAGE法分离并转移至醋酸纤维素膜上，以相应的单克隆抗体室温孵育4 h以检测目标蛋白。洗去一抗，以HRP连接的二抗室温孵育2 h，洗涤后以ECL试剂盒显示免疫反应条带。β-actin作为内参。

### 1.7 Real-time PCR检测A549/DDP细胞中MDR1、Bcl-2、survivin和PTEN的mRNA表达水平

取对数生长期的肿瘤细胞，1 μmol/L和5 μmol/L异长春花碱作用24 h后，用Trizol法提取各组总RNA，用real-time PCR试剂盒进行逆转录得到cDNA。MDR1上游引物序列：5'-AAAAAGATCAACTCGTACCACTC-3'，下游引物序列：5'-GCACAAAATACACCAACAA-3'；Bcl-2上游引物序列：5′- ACGGGGTGAACTGGGGGAGGA-3′，下游引物序列：5′- TGTTTGGGGCAGGCATGTTGACTT-3′；survivin上游引物序列：5′-ACAACCAAACCTCACACTACTG-3′，下游引物序列：5′-ATAGATCCCATTACAGACAGCG-3′；PTEN上游引物序列：5′-CTTTGTGCTGAAAGACATTATGAC-3′，下游引物序列：5′-GGCTTTGTCTTTATTTGCTTTGTC-3′；β-actin上游引物序列：5′-TGAGCGCGGCTACAGCTT-3′，下游引物序列：5′-TCCTTAATGTCACGCACGATTT-3′；94 ℃变性3 min后，按下述条件扩增40个循环：95 ℃ 5 s，65 ℃ 35 s，72 ℃ 60 s，循环后72 ℃延伸5 min。

### 1.8 A549/DDP细胞中NF-κB、Twist和Snail核转录活性的检测

根据试剂说明书的方法，每孔加入0.1 μg的NF-κB、Twist和Snail荧光报告质粒和0.02 μg对照质粒转染细胞，继续培养6 h，洗去未转入细胞的质粒，更换新鲜培养基，加入1 μmol/L和5 μmol/L异长春花碱作用24 h，以Dual-GloTM Luciferase assay system对两种萤光酶的活性进行检测。

### 1.9 统计学方法

实验数据以均数±标准差表示，使用SPSS 13.0软件进行分析。采用单因素方差分析（*One-way* *ANOVA*）进行比较，以*P* < 0.05表示差异具有统计学意义。

## 2 结果

### 2.1 异长春花碱增加A549/DDP细胞对顺铂的敏感性

MTS法检测结果显示，A549/DDP细胞经异长春花碱作用24 h后，细胞抑制率呈现剂量依赖性，1 μmol/L异长春花碱抑制率为3.7%，5 μmol/L异长春花碱抑制率为8.3%，分别为无毒浓度（细胞抑制率 < 5%）和低毒浓度（细胞抑制率 < 10%），故采用上述两种浓度进行实验（图 1）。A549/DDP细胞对顺铂的IC_50_为60.3 μmol/L，经1 μmol/L和5 μmol/L异长春花碱作用后，A549/DDP细胞对顺铂的敏感性明显升高，IC_50_分别为31.6 μmol/L和23.7 μmol/L（图 2），逆转倍数（RF）分别为1.91倍和2.54倍（*P* < 0.05），表明异长春花碱可提高A549/DDP细胞对顺铂的敏感性。

**1:**
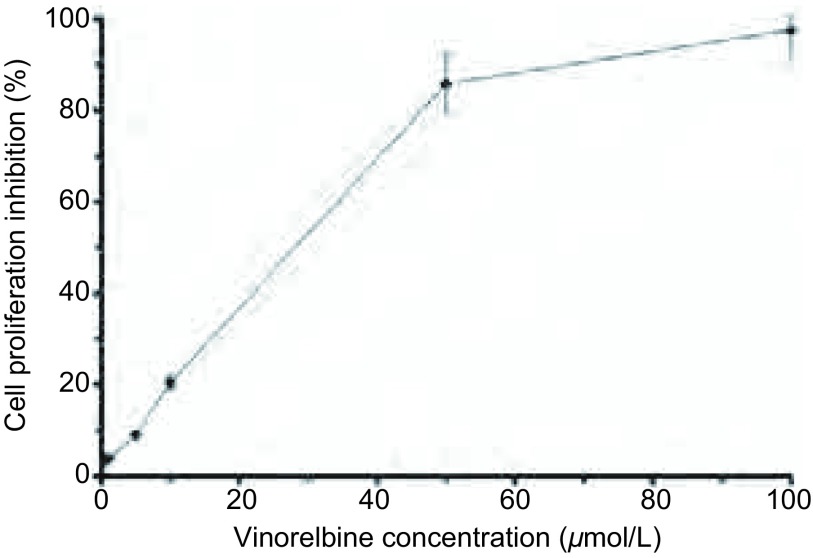
异长春花碱对A549/DDP细胞增殖的影响 The effect of Vinorelbine on A549/DDP cells proliferation. There was an increased cell proliferation inhibition with different concentration of Vinorelbine treatment in A549/DDP cell lines, the IC_50_ was 35.7 μmol/L. Bars indicate SD, *n*=5

**2:**
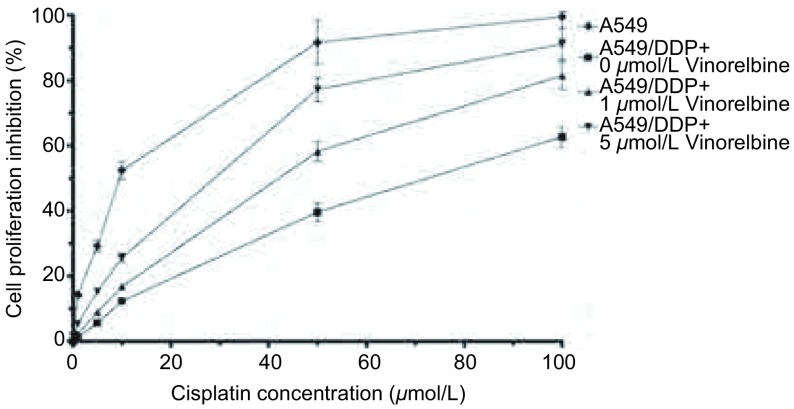
异长春花碱对A549/DDP细胞耐药性的逆转作用 Reversal effect of Vinorelbine on A549/DDP cells drug resistance. There was an increased cell proliferation inhibition with 1 μmol/L and 5 μmol/L Vinorelbine treatment in A549/DDP cell lines, the the IC_50_ of A549 was 9.8 μmol/L, the IC_50_ of A549/DDP without Vinorelbine treatment was 60.3 μmol/L, IC_50_ of A549/DDP with 1 μmol/L Vinorelbine treatment was 31.6 μmol/L, IC_50_ of A549/DDP with 5 μmol/L Vinorelbine treatment was 23.7 μmol/L, the sensitivity of cancer cells to cisplatin was increased by 1.91- and 2.54-folds respectively. Bars indicate SD, *n*=5

### 2.2 异长春花碱提高A549/DDP细胞胞内Rh-123浓度

促进细胞凋亡试验结果显示，1 μmol/L和5 μmol/L异长春花碱作用24 h后，A549/DDP细胞吸收荧光染料Rh-123的能力显著提高，与0 μmol/L组相比，细胞内Rh-123含量分别提高了1.93倍和2.95倍（图 3）。进一步的研究发现，1 μmol/L和5 μmol/L异长春花碱作用24 h后，A549/DDP细胞凋亡比例明显上升，与0 μmol/L组凋亡率11.4%相比，细胞凋亡率分别为25.7%和43.6%（图 4）。

**3:**
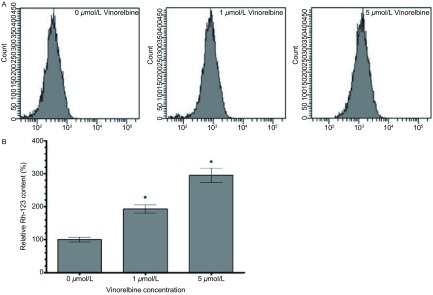
异长春花碱对A549/DDP细胞Rh-123蓄积的影响 The effect of Vinorelbine on the intracellular accumulation of Rh-123 in A549/DDP cells. A: The fow cytometry results in the effect of Vinorelbine on intra-cellular Rh-123 content of A549/DDP cells. B: The graph of the effect of Vinorelbine on the mean fluorescence intensity of Rh-123 in A549/DDP cells. The the content of Rh-123 was elevated 1.93- and 2.95-folds with 1 μmol/L and 5 μmol/L Vinorelbine treatment in A549/DDP cell lines compared with 0μmol/L Vinorelbine treatment. Data presented are Mean±SD, bars indicate SD, *n*=3, *: compared to 0 μmol/L group, *P* < 0.05

**4:**
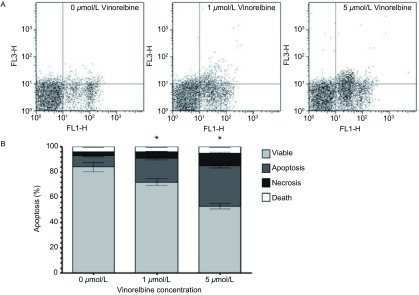
异长春花碱对A549/DDP细胞凋亡的影响 The effect of Vinorelbine on the apoptosis of A549/DDP cells. A: The fow cytometry results in the effect of Vinorelbine on inducing apoptosis of A549/DDP cells. B: The graph of the effect of Vinorelbine on the cells apoptosis rate of A549/DDP cells. The apoptosis rate was elevated 2.25- and 3.82-folds with 1 μmol/L and 5 μmol/L Vinorelbine treatment in A549/DDP cell lines compared with 0 μmol/L Vinorelbine treatment. Data presented are Mean±SD, bars indicate SD, *n*=3, *: compared to 0 μmol/L group, *P* < 0.05

### 2.3 异长春花碱调节A549/DDP细胞MDR1、Bcl-2、survivin和caspase-3/8的表达

为研究异长春花碱逆转A549/DDP耐药性，促进凋亡的机制，我们研究了多种耐药基因的表达。结果显示，1 μmol/L和5 μmol/L异长春花碱作用24 h后，MDR1、Bcl-2、survivin和caspase-3/8在蛋白水平的表达均显著下调，MDR1的mRNA表达下调43.5%和25.8%，Bcl-2的mRNA表达下调57.3%和34.1%，survivin的mRNA表达下调37.6%和12.4%（图 5）。

**5:**
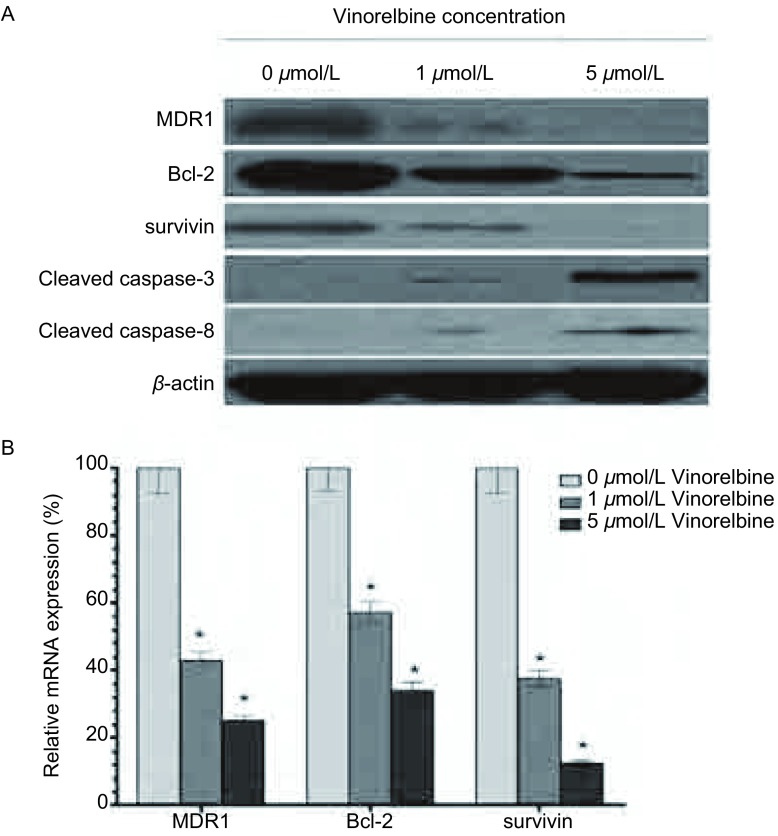
异长春花碱对A549/DDP细胞多药耐药基因表达的影响 The effect of Vinorelbine on the expression of drug resistance in A549/DDP cells. A: The effect of Vinorelbine on the protein expression of MDR1, Bcl-2, survivin and cleaved caspase-3/8 in A549/DDP cells. There was a downregulated expression of MDR, Bcl-2 and survivin, however, there was an upregulated expression of caspase-3/8 with Vinorelbine treatment. *β*-actin was as an internal control. B: The effect of Vinorelbine on the mRNA expression of MDR1, Bcl-2 and survivin in A549/DDP cells. Real-time assay showed that the mRNA expression of MDR1 was downregulated 43.5% and 25.8%, Bcl-2 was downregulated 57.3% and 34.1%, survivin was downregulated 37.6% and 12.4% with 1 μmol/L and 5 μmol/L Vinorelbine treatment in A549/DDP cell lines compared with 0 μmol/L Vinorelbine treatment. *β*-actin was as an internal control. Data presented are Mean±SD, bars indicate SD, *n*=5, *: compared to 0 μmol/L group, *P* < 0.05

### 2.4 异长春花碱抑制Akt的磷酸化，上调PTEN表达，下调NF-κB、Twist和Snail的转录活性

为研究异长春花碱调节上述耐药和凋亡相关蛋白的机制，我们重点研究了一些关键信号通路的变化。Western blot显示，1 μmol/L和5 μmol/L异长春花碱作用24 h后，肿瘤细胞Akt的磷酸化水平下降，PTEN蛋白表达上调，PTEN的mRNA表达上调183.4%和154.2%，而用报告基因系统评价NF-κB、Twist和Snail这些转录因子的活性时，结果显示，这些因子的转录活性均明显下降，与0 μmol/L组相比，NF-κB转录活性下降53.2%和34.5%，Twist转录活性下降61.4%和33.5%，Snail转录活性下降57.8%和18.7%（图 6），说明异长春花碱逆转A549/DDP细胞顺铂耐药性可能通过上调PTEN表达，抑制AKT/NF-κB信号路径活性，进而抑制下游转录因子Twist和Snail转录活性，进一步调节下游基因表达。

**6:**
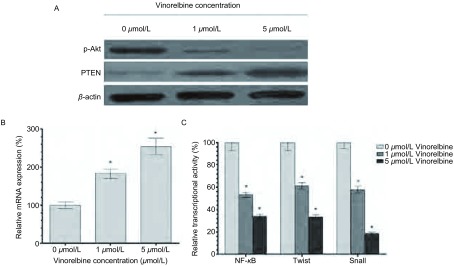
异长春花碱对A549/DDP细胞信号转导路径的影响 The effect of Vinorelbine on the signal transduction in A549/DDP cells. A: The effect of Vinorelbine on the expression of p-Akt and PTEN in A549/DDP cells with Vinorelbine treatment. *β*-actin was as an internal control. B: The effect of Vinorelbine on the mRNA expression of PTEN in A549/DDP cells. The mRNA expression of PTEN was upregulated183.4% and 154.2% with 1 μmol/L and 5 μmol/L Vinorelbine treatment in A549/DDP cell lines compared with 0 μmol/L Vinorelbine treatment. *β*-actin was as an internal control. Data presented are Mean±SD, bars indicate SD, *n*=5, *: compared to 0 μmol/L group, *P* < 0.05. C: The effect of Vinorelbine on the transcriptional activity of NF-*κ*B, Twist and Snail in A549/DDP cells. The transcriptional activities of NF-κB was downregulated 53.2% and 34.5%, Twist was downregulated 61.4% and 33.5%, and Snail was downregulated 57.8% and 18.7% with 1 μmol/L and 5 μmol/L Vinorelbine treatment in A549/DDP cell lines compared with 0 μmol/L Vinorelbine treatment. Data presented are Mean±SD, bars indicate SD, *n*=5, *: compared to 0μmol/L group, *P* < 0.05

## 3 讨论

肺癌是最常见的恶性肿瘤之一，铂类药物是一类广泛使用的化疗药物，在多种恶性肿瘤的治疗中表现出很好的效果。但肺癌治疗过程中肿瘤细胞发展出来的耐药性，往往导致治疗失败，寻找可运用于临床抵抗耐药性的新药，是提高患者临床受益的迫切需要。

肿瘤细胞对化疗药物产生耐受性的机制之一是过表达MDR1等多药耐药蛋白，增加药物的外排，降低药物在细胞内蓄积和在药靶部位的有效浓度^[5-7]^。在本研究中，我们发现异长春花碱可增加A549/DDP细胞对顺铂的敏感性，并具有剂量依赖性。我们利用流式细胞术检测了肿瘤细胞对荧光染料Rh-123吸收的变化，结果显示，异长春花碱水平越高，则胞内Rh-123的含量越高。Rh-123是MDR1底物，胞内Rh-123的含量提高可间接说明肿瘤细胞对化疗药物的外排作用降低。进一步的WB和real-time PCR研究显示，MDR1的蛋白和mRNA表达水平均下降，与Rh-123的研究结果一致。

除MDR1以外，凋亡相关基因也可以介导肿瘤细胞对顺铂的耐受。半胱天冬酶8（caspase-8）是细胞凋亡过程中的重要效应物质，激活后可释放到胞质中启动caspase的级联反应，激活下游的半胱天冬酶3（caspase-3），导致细胞凋亡。Bcl-2和survivin是肿瘤细胞中重要的凋亡抑制基因，已经被证明在耐药肿瘤细胞中过表达，可通过阻断凋亡终末效应酶caspase-3活性抑制细胞凋亡。本研究的流式细胞分析结果显示，异长春花碱可促进细胞的凋亡，并发现异长春花碱可下调Bcl-2和survivin表达，上调caspase-3/8表达。因此，我们认为异长春花碱是通过下调凋亡抑制基因，上调促凋亡基因表达实现对肿瘤细胞凋亡的调控作用。

*PTEN*是一种肿瘤抑制基因，可通过抑制Akt磷酸化，下调Akt活性，阻断Akt下游信号转导路径及，调节下游基因表达^[8, 9]^。我们发现异长春花碱可上调PTEN表达，抑制Akt磷酸化和NF-κB转录活性。Twist和Snail是NF-κB的下游靶基因，也是重要的促肿瘤转录因子，可通过调节下游基因在肿瘤耐药产生过程中发挥重要作用，我们的结果也证明长春花碱可下调Twist和Snail的转录活性。

综上所述，我们认为长春花碱逆转A549/DDP顺铂耐药性可能与增加肿瘤细胞药物蓄积，诱导凋亡有关，调节PTEN/AKT/NF-κB信号路径活性，进而调节下游Twist和Snail等转录因子活性和耐药相关基因表达，可能是其作用机制的中心环节。

## References

[1] Dela Cruz CS, Tanoue LT, Matthay RA (2011). Lung cancer: epidemiolog y, etiology, and prevention. Clin Chest Med.

[2] Liu MY, Li CH, Yan A (2010). cDNA microarray technique on screening multi-drug resistance-related genes of human non-small cell lung cancer. Zhongguo Fei Ai Za Zhi.

[3] Liu XY, Zhang SM, Xu SM (2009). Expression and clinical significance of LRP and MRP in non-small cell lung cancer tissues by bronchoscopy biopsy. Zhongguo Fei Ai Za Zhi.

[4] Mi YJ, Liang YJ, Huang HB (2010). Apatinib (yn968d1) reverses multidrug resistance by inhibiting the efflux function of multiple atp-binding cassette transporters. Cancer Res.

[5] Chen YT, Feng B, Chen LB (2012). Update of research on drug resistance in small cell lung cancer chemotherapy. Asian Pac J Cancer Prev.

[6] Orlandi F, Coronnello M, Bellucci C (2013). New structure-activity relationship studies in a series of N, N-bis (cyclohexanol) amine aryl esters as potent reversers of P-glycoprotein-mediated multidr ug resistance (MDR). Bioorg Med Chem.

[7] Munić V, Kelnerić Z, Mikac L (2010). Differences in assessment of macrolide interaction with human MDR1 (ABCB1, P-gp) using rhodamine-123 efflux, ATPase activity and cellular accumulation assays. Eur J Pharm Sci.

[8] Tanaka K, Babic I, Nathanson D (2011). Oncogenic EGFR signaling activates an mTORC2-NF-κB pathway that promotes chemotherapy resistance. Cancer Discov.

[9] Song W, Jiang R, Zhao CM (2012). Role of integrin-linked kinase in multi-drug resistance of human gastric carcinoma SGC7901/DDP cells. Asian Pac J Cancer Prev.

